# Modulation of Immunity by Lymphatic Dysfunction in Lymphedema

**DOI:** 10.3389/fimmu.2019.00076

**Published:** 2019-01-29

**Authors:** Yinan Yuan, Valeria Arcucci, Sidney M. Levy, Marc G. Achen

**Affiliations:** ^1^Tumour Angiogenesis and Microenvironment Program, Peter MacCallum Cancer Centre, Melbourne, VIC, Australia; ^2^Sir Peter MacCallum Department of Oncology, University of Melbourne, Parkville, VIC, Australia

**Keywords:** immune function, inflammation, lymphedema, regulatory T cells, T-helper cells

## Abstract

The debilitating condition known as secondary lymphedema frequently occurs after lymphadenectomy and/or radiotherapy for the treatment of cancer. These therapies can damage lymphatic vessels leading to edema, fibrosis, inflammation and dysregulated adipogenesis, which result in profound swelling of an affected limb. Importantly, lymphedema patients often exhibit impaired immune function which predisposes them to a variety of infections. It is known that lymphadenectomy can compromise the acquisition of adaptive immune responses and antibody production; however the cellular mechanisms involved are poorly understood. Here we discuss recent progress in revealing the cellular and molecular mechanisms underlying poor immune function in secondary lymphedema, which has indicated a key role for regulatory T cells in immunosuppression in this disease. Furthermore, the interaction of CD4^+^ T cells and macrophages has been shown to play a role in driving proliferation of lymphatic endothelial cells and aberrant lymphangiogenesis, which contribute to interstitial fluid accumulation in lymphedema. These new insights into the interplay between lymphatic vessels and the immune system in lymphedema will likely provide opportunities for novel therapeutic approaches designed to improve clinical outcomes in this problematic disease.

## Introduction

The lymphatic system is a highly structured vascular network, important for interstitial fluid homeostasis, immune surveillance and lipid absorption, which consist of distinct types of lymphatic vessels. Interstitial fluid is absorbed by highly permeable initial lymphatics and transported by lymphatic pre-collectors to lymphatic collectors, which converge to form lymphatic trunks that ultimately transport lymph to the venous system via lymphatic ducts. Each type of lymphatic vessel is anatomically specialized for its function (1), but all lymphatic vessels share the feature of being lined by a single layer of lymphatic endothelial cells (LECs). Lymphatic vessels can undergo a variety of remodeling processes in development and disease, including lymphangiogenesis (the growth of new lymphatic vessels), which have important implications for lymphatic biology and immune function (2). Much progress has been made over recent years in defining the effects of lymphatic remodeling, lymphangiogenesis and LECs on immune function, particularly in the setting of cancer [for example see (3–5)]. More specifically, the establishment of tumor-associated immunity is thought to depend on lymphatic vessel remodeling and drainage. Further, there is emerging evidence that LECs are important for the maintenance of peripheral tolerance, modulating effector T cell responses and influencing leukocyte function (6). Here we review the role of lymphatic vessels in modulating immunity in secondary lymphedema, a prevalent condition caused by lymphatic dysfunction, which involves remodeling of lymphatic vessels and compromised immune function.

## Secondary Lymphedema: Clinical Aspects and Pathophysiology

Secondary lymphedema is a chronic disease characterized by the accumulation of interstitial fluid in tissues due to damaged lymphatic vessels, leading to swelling, and dysfunction of limbs (7). It is an acquired condition that is etiologically distinct from primary, or hereditary, lymphedema, which is a rare disease caused by intrinsic abnormalities of lymphatic function due to defects in genes involved in the growth and development of the lymphatic vasculature (8). Secondary lymphedema is a slow but progressive condition which can be caused by trauma, infection and inflammation. Globally, the most common cause is lymphatic filariasis, due to lymphatic vascular invasion by filarial nematodes, which has been estimated to afflict 68 million people in 73 countries worldwide (9). However, disruption of the lymphatic vasculature due to surgical interventions (e.g., lymphadenectomy) and/or radiotherapy for breast cancer is the most common cause in the developed world (7), with an incidence of lymphedema of 21% among women who were diagnosed with breast cancer (10). The condition can occur not only in breast cancer patients, but in patients with any cancer types which require lymph node dissection or radiotherapy treatment, such as head and neck, genitourinary and gynecological cancers, and melanoma (11). The onset of secondary lymphedema can be highly variable and has been reported to occur immediately postoperatively or up to 30 years post-treatment in the context of breast cancer, and it is not clear what determines a patient's predisposition to develop the disease. Historically, secondary lymphedema has been considered underdiagnosed, and robust epidemiological data have been scant, however, it is clearly a relatively prevalent condition with between two and five million people estimated to suffer from it in the United States (12).

Secondary lymphedema can be highly debilitating both physically and psychologically for patients due to the reduced quality of life associated with limb discomfort, anxiety, depression, sexual dysfunction, and social isolation. Current treatment choices for lymphedema include massage, manual lymph drainage (13), remedial exercise (14), compression bandaging (15), electrophysical modalities (including low-level laser therapy and electrical stimulation) (16), elevation techniques, exercise programs, and dietary/weight loss interventions (17, 18). There are also a range of surgical options such as liposuction (19), various forms of vascular anastomosis (20), lymph node transplantation (21) and other regional tissue transfer procedures. Unfortunately these treatments have not proven to be reliably curative as they have limited efficacy in controlling the disease, and many do not address the cause. Notably, there are no molecular-based therapies for the condition although therapeutics targeting inflammation (ketoprofen) (22) or promoting lymphangiogenesis [Lymfactin and Ubenimex also known as Bestatin (23, 24)] are being tested for treatment of lymphedema in clinical trials programs-see ClinicalTrials.gov for further information about these trials (ClinicalTrials.gov identifiers: NCT02257970, NCT02700529, and NCT02994771). Given the absence of curative treatment options and prevalence of the condition, secondary lymphedema is considered an important unmet clinical need in medicine.

The pathological features of secondary lymphedema include edema, inflammation, dermal fibrosis and formation of fat tissue, and patients often exhibit impaired immune function predisposing them to a variety of infections (25). These features are thought to further restrict lymphatic function in lymphedematous tissue thereby establishing a vicious pathophysiological cycle (26). The types of infections observed in secondary lymphedema include cellulitis involving the deeper dermis and subcutaneous fat, erysipelas involving the superficial dermis and lymphangitis involving the superficial dermal lymphatics. Soft-tissue infections associated with secondary lymphedema can lead to sepsis and, on occasions, death (27). Therefore, patients can require lifelong prophylactic antibiotic therapy. Given the clinical management of lymphedema-associated infections can be highly problematic, it is important to understand how the immune response is impaired by the lymphatic injury which underlies secondary lymphedema. Such understanding could provide opportunities for prevention or improved treatment of secondary lymphedema.

## Immunological Vulnerability of Secondary Lymphedema

**Figure 1:**
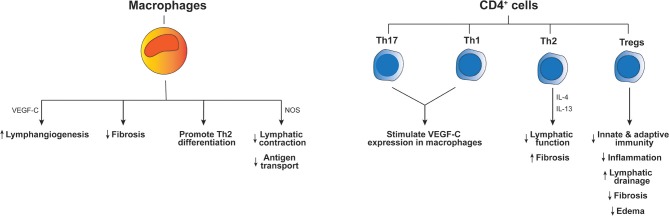
Effects of immune cells on the pathogenesis of lymphedema. The diagram indicates how different immune cell types influence inflammation and other aspects of lymphedema pathology. Key molecular mediators produced by these cells types, which have been reported to drive the outcomes listed at the bottom of the figure, are indicated next to the long arrows. “Th” denotes T-helper cells, “Tregs” regulatory T cells, “IL” interleukin and “NOS” inducible nitric oxide synthase.

Extensive clinical literature and experience has made it clear that lymphedematous tissue is immunologically vulnerable. Not only infections, but also neoplasms and immune-related disorders, such as neutrophilic dermatosis and toxic epidermal necrolysis, occur more frequently than in normal tissue [for example see (28)]. Chronic secondary lymphedema is typically characterized by an altered abundance of immune cells. Clinical studies have demonstrated increased numbers of lymphocytes, plasma cells, macrophages, dendritic cells, and neutrophils in the affected skin and subdermal tissue of lymphedema patients (29, 30). Such immune cell populations can be important for development of lymphedema, for example a CD4^+^ cell inflammatory response and T-helper 2 (Th2) cell differentiation can contribute to key pathological changes including fibrosis and lymphatic dysfunction (Figure 1) (31). The accumulation of macrophages and lymphocytes in lymphedematous tissue can be induced by lymphatic fluid stasis in animal models of lymphedema (32). Importantly, there is evidence from animal models that lymphatic vascular defects can be associated with inadequate humoral immunity (33) which is consistent with clinical studies in lymphedema patients which showed that vaccination in lymphedematous tissue was associated with significantly decreased antibody titres (34).

Lymphedematous tissue constitutes a highly abnormal environment from the perspective of immune function. It is to be expected that chronic lymph stasis in lymphedema would impair local immune surveillance by restricting the trafficking of immunocompetent cells in lymphedematous tissues. Further, the irregular accumulation of immune mediators (cytokines and chemokines) in lymphedematous tissue could be an initiating factor promoting activation of LECs and immune cells (35). It is well-known that lymphatics can respond to immune mediators produced by macrophages, including TNF-α, IL-1β, and IFNγ (36), and are able to produce immune mediators which regulate macrophage function such as IL-6 and CCL21 (35, 37). It has also been shown that macrophages produce inducible nitric oxide synthase which causes a reduction of lymphatic vessel contraction (38) and thus may contribute to accumulation of tissue fluids and impairment of antigen transport to lymph nodes in lymphedema (Figure 1). In summary, it is likely that lymphatic activation perpetuates abnormal activation of macrophages, and visa-versa, which could contribute to immune dysfunction and abnormal inflammation in lymphedema. Nevertheless, the cellular mechanisms which cause immune deficits in lymphedema have begun to emerge only recently.

## Mechanisms of Immune Dysfunction in Secondary Lymphedema

A recent study exploring the function of T cells in lymphedema demonstrated a major increase in regulatory T cells (Tregs) in the lymphedematous extremity, compared to contralateral control tissue, in patients with breast cancer-related lymphedema (39). This finding was replicated in a mouse model of axillary lymph node dissection which showed increased infiltration of both CD4^+^ T cells and Tregs. In this model, it was shown that Treg proliferation was localized to the tissue distal to the area of lymphatic injury caused by the surgery. Further analyses suggested that the Tregs downregulated local tissue inflammation post-lymphatic injury, and inhibited acquisition of T-cell-mediated immune responses (39). The loss of draining lymph nodes was also thought to diminish these responses. In addition, Tregs impaired bacterial phagocytosis, regulated humoral responses and compromised dendritic cell activation in this model after lymphatic injury (39). Overall, Tregs impaired both innate and adaptive immune responses, and depletion of these cells restored immune-mediated responses, indicating an important role for these cells in local immunosuppression in lymphedema (39) (Figure 1).

Immune function has been studied in transgenic mice expressing a soluble form of the lymphangiogenic receptor VEGFR-3 in skin (*K14-VEGFR-3-Ig* mice). These mice lack small dermal lymphatic vessels, develop lymphedema, and provide a model in which to monitor immune responses in the setting of lymphatic insufficiency (40). These mice produced lower antibody titres in response to dermal immunization, which was not due to compromised function of B cells, but was thought to be due to physiological differences in antigen transport to draining lymph nodes (33). T cell responses to dermal vaccination were delayed in these mice, although these responses were nevertheless robust. T-cell-mediated contact hypersensitivity (CHS) responses were strong, but the ability of these transgenic mice to induce CHS tolerance in the skin was impaired (33). The mice also exhibited hallmarks of autoimmunity, including antibody deposits in the skin, which supports the concept that lymphatic drainage to lymph nodes is important for maintaining immune tolerance against peripheral antigens. These findings provide mechanistic insight into how compromised lymphatic drainage in lymphedema plays a role in regulating humoral immunity and peripheral tolerance (33).

The effect of re-introducing lymph nodes, post-lymphatic damage, on immune responses and development of secondary lymphedema was monitored by Huang et al. in a mouse model of lymphatic ablation and popliteal lymph node dissection (41). Lymph node transplantation in this model led to a decreased accumulation of perilymphatic inflammatory cells, increased dendritic cell trafficking from the periphery to the inguinal node, and markedly improved adaptive immune responses. These changes were accompanied by decreases in hindlimb swelling and fibroadipose tissue deposition, as well as a pronounced lymphangiogenic response. The findings from this model may have clinical relevance for improving immune function post-lymphatic damage, given lymph node transfer is being used in human patients and is being developed in animal models in combination with lymphangiogenic growth factor therapy (42–44).

## Effects of Immune Cells on Lymphedema Pathophysiology

The involvement of CD4^+^ T cells in lymphedema pathogenesis was studied by Ogata et al. who employed a mouse model of lymphedema based on ligating the major collecting lymphatic vessels in the skin of the abdomen and removing the associated axillary lymph node (30). This model exhibited excessive generation of immature lymphatic vessels that was essential for the early emergence of edema and the subsequent development of lymphedema pathology. CD4^+^ T cells interacted with macrophages to promote lymphangiogenesis in this model, and both lymphangiogenesis and edema were reduced in macrophage-depleted or CD4^+^ T-cell-deficient mice. From a mechanistic perspective, Th1 and Th17 cells activated macrophages to produce the lymphangiogenic growth factor VEGF-C, which likely drove the aberrant lymphangiogenesis. Inhibition of this mechanism suppressed both early lymphangiogenesis and development of lymphedema (30). Macrophages have also been reported to restrict fibrosis as depletion of these cells in a mouse model of secondary lymphedema significantly increased fibrosis, and impaired lymphatic transport, decreased VEGF-C expression and promoted Th2 differentiation (45). Th2 cells may also be involved in lymphedema pathogenesis as neutralization of two cytokines produced by these cells, IL-4 and IL-13, in a mouse model of secondary lymphedema promoted lymphatic function and restricted fibrosis (31). The role of CD4^+^ T cells was also studied in a mouse model of secondary lymphedema by use of adoptive transfer techniques in CD4-deficient mice that underwent excision of skin and lymphatics in the tail or dissection of popliteal lymph nodes (46). This study revealed naïve CD4^+^ T cells were activated in skin-draining lymph nodes and then migrated to lymphedematous skin. These activated cells promoted fibrosis and inflammation, and inhibited lymphangiogenesis and lymphatic function. Importantly, use of a sphingosine-1-phosphate receptor modulator to block release of T cells from lymph nodes prevented lymphedema in the mouse tail model employed in this study (46). It is now clear that CD4^+^ T cells play a major role in the development of secondary lymphedema (Figure 1), at least in animal models, although the effect of these cells on lymphangiogenesis in lymphedema differed in different mouse models and therefore requires further clarification.

In a separate study, RNA sequencing of lymphedematous mouse skin suggested an upregulation of many T cell-related networks (47). More specifically, upregulation of Foxp3, a transcription factor specifically expressed by Tregs, indicated a potential role for these cells in lymphedema, consistent with findings discussed in the previous section. While global deletion of CD4^+^ cells restricted lymphedema development in the mouse tail lymphedema model used in this study, targeted depletion of Tregs led to exacerbated edema associated with increased infiltration of immune cells and a mixed Th1/Th2 cytokine profile (47). Conversely, expansion of Tregs in the mouse model restricted lymphedema development. Therapeutic use of adoptively transferred Tregs upon lymphedema establishment reversed the major hallmarks of lymphedema such as edema, fibrosis and inflammation, and promoted lymphatic drainage (47). These findings on the role of Tregs are supported by the study of Garcia Nores et al. which showed that depletion of Tregs up-regulated local tissue inflammation after lymphatic injury (39). However, this study also showed that Tregs can locally impair adaptive immunity and clearance of bacteria after lymphatic injury. While it is clear that the number of Tregs in lymphedematous tissue is increased compared to normal tissue, the functional significance of these cells for development of lymphedema may differ depending on the relative importance of inflammation vs. adaptive immunity in the lymphedema model employed. This issue needs to be considered when assessing if Treg application could be a potential new therapeutic approach for treating lymphedema.

## Concluding Remarks

Recent studies using animal models and clinical samples have established that immune function is significantly compromised in secondary lymphedema, and demonstrated that a variety of T-cell-related networks are up-regulated in this condition. Tregs, in particular, are increased in abundance in lymphedematous tissue and are thought to compromise immune function in this disease by promoting immunosuppression, although they can make a positive contribution by reducing the degree of inflammation. Further analysis of Treg function in secondary lymphedema is required to establish whether or not modulating the levels or function of these cells could be beneficial for prevention or treatment of this condition. This may need to be pursued in large animal models, as opposed to mice, to give a clearer picture of how targeting immune cells might be beneficial for lymphedema patients.

## Author Contributions

YY and MA conceived the topic and outlined the paper. YY, VA, SL, and MA wrote the paper.

### Conflict of Interest Statement

MA is a shareholder in Opthea Ltd. and an Inventor on patents assigned to Vegenics Ltd. The remaining authors declare that the research was conducted in the absence of any commercial or financial relationships that could be construed as a potential conflict of interest.
